# *ATP2C2* and *DYX1C1* are putative modulators of dyslexia‐related MMR

**DOI:** 10.1002/brb3.851

**Published:** 2017-10-18

**Authors:** Bent Müller, Gesa Schaadt, Johannes Boltze, Frank Emmrich, LEGASCREEN Consortium, Michael A. Skeide, Nicole E. Neef, Indra Kraft, Jens Brauer, Angela D. Friederici, Holger Kirsten, Arndt Wilcke

**Affiliations:** ^1^ Fraunhofer Institute for Cell Therapy and Immunology Leipzig Germany; ^2^ Department of Neuropsychology Max Planck Institute for Human Cognitive and Brain Sciences Leipzig Germany; ^3^ Department of Psychology Humboldt‐Universität zu Berlin Berlin Germany; ^4^ Department of Medical Cell Technology Fraunhofer Research Institution for Marine Biotechnology Lübeck Germany; ^5^ Institute for Medical and Marine Biotechnology University of Lübeck Lübeck Germany; ^6^ Institute for Medical Informatics Statistics and Epidemiology University of Leipzig Leipzig Germany; ^7^ LIFE—Leipzig Research Center for Civilization Diseases University of Leipzig Leipzig Germany

**Keywords:** auditory discrimination, child, dyslexia, electroencephalography, eQTL, genetic predisposition to disease, German language, intermediate phenotype, mismatch negativity, single‐nucleotide polymorphism

## Abstract

**Background:**

Dyslexia is a specific learning disorder affecting reading and spelling abilities. Its prevalence is ~5% in German‐speaking individuals. Although the etiology of dyslexia largely remains to be determined, comprehensive evidence supports deficient phonological processing as a major contributing factor. An important prerequisite for phonological processing is auditory discrimination and, thus, essential for acquiring reading and spelling skills. The event‐related potential Mismatch Response (MMR) is an indicator for auditory discrimination capabilities with dyslexics showing an altered late component of MMR in response to auditory input.

**Methods:**

In this study, we comprehensively analyzed associations of dyslexia‐specific late MMRs with genetic variants previously reported to be associated with dyslexia‐related phenotypes in multiple studies comprising 25 independent single‐nucleotide polymorphisms (SNPs) within 10 genes.

**Results:**

First, we demonstrated validity of these SNPs for dyslexia in our sample by showing that additional inclusion of a polygenic risk score improved prediction of impaired writing compared with a model that used MMR alone. Secondly, a multifactorial regression analysis was conducted to uncover the subset of the 25 SNPs that is associated with the dyslexia‐specific late component of MMR. In total, four independent SNPs within *DYX1C1* and *ATP2C*2 were found to be associated with MMR stronger than expected from multiple testing. To explore potential pathomechanisms, we annotated these variants with functional data including tissue‐specific expression analysis and eQTLs.

**Conclusion:**

Our findings corroborate the late component of MMR as a potential endophenotype for dyslexia and support tripartite relationships between dyslexia‐related SNPs, the late component of MMR and dyslexia.

## 1 INTRODUCTION

Dyslexia is a learning disorder affecting the acquisition of reading and spelling skills. According to the Diagnostic and Statistical Manual of Mental Disorders: DSM‐V (American Psychiatric Association, 2013), reading as well as spelling impairments belong to the category of specific learning disorders. They can occur independently or in combination.

Dyslexia has a prevalence of 5% among German‐speaking individuals (Moll, Kunze, Neuhoff, Bruder, & Schulte‐Körne, 2014). The heritability was estimated at 50%–70% (de Kovel et al., 2004; Harlaar, Spinath, Dale, & Plomin, 2005; Shaywitz et al., 1998), but only a small proportion of the genetic basis of dyslexia has been uncovered. Linkage studies pointed at nine chromosomal regions (termed DYX1 to 9) and subsequent association studies identified several dyslexia‐related genes within these regions, for example, *DYX1C1*,* DCDC2*,* KIAA0319*, and *ROBO1*. Moreover, associations with genes outside these regions such as *CMIP*,* CNTNAP2,* and *FOXP2* have also been repeatedly reported (Peter et al., 2011; Scerri et al., 2011; Vernes et al., 2008). A frequent comorbidity of dyslexia is specific language impairment (SLI). For example, McArthur and colleagues (McArthur, Hogben, Edwards, Heath, & Mengler, 2000) demonstrated that more than 50% of children with SLI were also diagnosed with dyslexia. This strong overlap corroborates the hypothesis of a shared genetic background of reading and language abilities. Indeed, the genetic correlation was estimated at between 0.67 and 1.00 (Plomin & Kovas, 2005). Thus, it is plausible to consider candidate single‐nucleotide polymorphisms (SNPs) reported to be associated with SLI also as relevant candidate SNPs for dyslexia and dyslexia‐related processes. Genes with already reported associations to both, SLI and dyslexia, include *FOXP2* (Lai, Fisher, Hurst, Vargha‐Khadem, & Monaco, 2001; Wilcke et al., 2012), *KIAA0319* (Cope et al., 2005; Newbury et al., 2011), *CNTNAP2* (Newbury et al., 2011; Peter et al., 2011; Vernes et al., 2008), and *CMIP* (Newbury et al., 2009; Scerri et al., 2011).

However, knowledge regarding specific pathomechanisms translating genetic risk variants into a dyslexic phenotype is still very limited. Endophenotypes are a common concept for describing pathomechanistical processes. Endophenotypes are defined as measurable, phenotypic components contributing to disease‐phenotype and found along the path from genes to the disease‐phenotype (Gottesman & Gould, 2003). Certain dyslexia‐related potential endophenotypes affected by genetic risk variants have been reported in neuroimaging studies analyzing specific hemodynamic brain activation patterns. Exemplarily, SNPs in *FOXP2* were associated with fMRI activation in the left inferior frontal and precentral gyri, whereas SNP rs17243157 in *THEM2* was associated with asymmetry in the functional activation of the superior temporal sulcus (Pinel et al., 2012). Furthermore, Darki, Peyrard‐Janvid, Matsson, Kere, and Klingberg (2012) reported gray and white matter variation to be linked with variants within *DYX1C1*,* DCDC2,* and *KIAA0319* dyslexia candidate genes, while Scerri et al. (2012) showed white matter variation to be linked with variants within *MRPL19/C2ORF3*. These associations between dyslexia candidates and brain structure are in line with findings from MRI studies, where structural gray and white matter alterations were associated with dyslexia‐relevant traits (Klingberg et al., 2000; Kraft et al., 2015).

Another potential neurophysiological endophenotype for dyslexia refers to automatic responses being observable in a specific component of the auditory event‐related potential (ERP). This is called mismatch negativity or, more generally spoken, mismatch response (MMR) (Näätänen, Gaillard, & Mäntysalo, 1978). Altered MMRs are reported for individuals with dyslexia and SLI, and are assumed to be associated with deficient phonological processing (Lovio, Näätänen, & Kujala, 2010; Schulte‐Körne, Deimel, Bartling, & Remschmidt, 1998, 2001). Indeed, first evidence for genetic variants affecting the late component of MMR was reported in a previous genome‐wide association study (GWAS) for common variants (Roeske et al., 2011) and, subsequently, for certain rare variants (Czamara et al., 2011).

In this study we investigated the neuro‐functional implications of dyslexia candidate genes. Specifically, we wanted to uncover the relationship between dyslexia‐related phenotypes and the late component of MMR on the genetic level. To this end, we identified 25 independent SNPs from 10 genes previously reported to be associated with dyslexia or dyslexia‐related phenotypes in at least two studies. Details on the selected SNPs can be found in Materials and Methods. Additionally, we investigated two common SNPs previously reported to be associated with the late component of MMR. These SNPs were tested regarding a possible association with the late component of the MMR in a sample of 67 children.

## 2 MATERIALS AND METHODS

### 2.1 Participants

Sixty‐seven children (37% male subjects), mean age 9.63 years (*SD* = 0.53), participated in this study. All children were monolingual German, attending primary school (grade 3 and 4) without any history of hearing impairment or neurological disorders. Twelve children (10 male subjects) were diagnosed with attention deficit disorder (ADD).

German individuals with dyslexia are more likely to show spelling difficulties than reading difficulties (Landerl, Wimmer, & Frith, 1997; Wimmer, 1996). Therefore, the DERET (Deutscher Rechtschreibtest; German Spelling Test) (Stock & Schneider, 2008) was used to specify participants’ spelling abilities. The DERET qualitatively and quantitatively assesses the orthographic abilities of primary students in accordance with German curricula. Dictations mirror children's ability to use German phoneme‐grapheme‐correspondence as well as orthographic rules (see Supporting Information 1 for details).

In addition, we assessed nonverbal intelligence using the German version of the Kaufmann‐Assessment Battery for Children (K‐ABC) (Kaufman, Kaufman, Melchers, & Preuß, 2009). Importantly, no child had an IQ (i.e., nonverbal) below the critical threshold of 85. Descriptive statistics for demographic and psychometric (DERET, K‐ABC) variables are presented in Table 1 and further details can be found in Table S1.

**Table 1:** Demographic and psychometric information on 10‐year‐old children

*N*	67
Handedness (right:left)	61:6
Nonverbal intelligence (mean IQ)	111.29 ± 9.60
DERET (mean PR)	42.42 ± 29.40
DERET (PR < 10:PR > 10)	14:53
DERET ≤ 10 (boys:girls)	10:4

*N*, number of participants; number in brackets, standard deviations; PR, percentile rank; DERET, Deutscher Rechtschreibtest (German spelling test).

Parents of participating children were reimbursed (€7.00 per hour). The study followed American Psychological Association (APA) standards in accordance with the declaration of Helsinki from 1964 (World Medical Organization, 1996) and was approved by the medical faculty of the University Leipzig. Children and their parents were informed both orally and in writing about the procedures and parents had to provide written consent for their children's enrolment.

### 2.2 Stimulus material

In order to analyze auditory speech discrimination capabilities by means of MMR, we conducted a passive oddball paradigm, where participants were presented with a frequently occurring standard syllable, occasionally replaced by a deviant syllable. We used the syllables /pa/ (266 ms in length) and /ga/ (409 ms in length), which were recorded by a native German speaker. The stimuli were recorded with a 16‐bit sampling rate and digitized at 44.1 kHz.

### 2.3 EEG testing

The children were seated in a comfortable chair in an electrically and acoustically shielded electroencephalography (EEG) cabin. Auditory stimuli were presented binaurally via the tannoy with an intensity of 64 dB sound pressure level (SPL). During the presentation, the children watched a silent video of “the little mole”, a popular children's cartoon (http://www.imdb.com/title/tt0841927/), on a small video screen in front of them. This was done to prevent extreme eye movements and boredom. We used a two‐block design because of the different duration characteristics of the two syllables. The syllable/ga/was used as the standard and the syllable/pa/as the deviant in one block; and vice versa in the other block. The order of the two blocks was counterbalanced across the children. Within one block, 600 stimuli were presented with 510 standard (85%) and 90 deviant stimuli (15%). We pseudorandomized the presentations of the deviant stimuli so that at least two standard stimuli were presented in between the deviant stimuli. The inter‐stimulus‐interval (ISI) between two stimuli (offset to onset) varied between 1,450 and 1,750 ms related to the different duration characteristics of the syllables. This is a time range during which the MMR is still elicited (Sams, Hari, Rif, & Knuutila, 1993). The experiment lasted for 45 min and the total procedure for about 90 min.

### 2.4 EEG data recording

The EEG was continuously recorded from Ag/AgCl cap‐mounted electrodes (Easy Cap GmbH, Germany) in accordance to the 10–20 International System of Electrode Placement and using the QRefa Acquisition Software, Version 1.0 beta (Max Planck Institute for Human Cognitive and Brain Sciences, Leipzig, Germany). Electrode sites were the following: F7, F3, FZ, F4, F8, FC3, FC4, T7, C3, CZ, C4, P7, CP5, CP6, T8, P3, PZ, P4, P8, O1, O2, A1, and A2. During the recordings, the electrodes were referenced to CZ, and an additional electrode placed at FP1 served as common ground. Electrooculograms (EOG) were recorded bipolarly from supraorbital and infraorbital sites at the right eye, as well as from electrodes located at the respective outer canthus. Electrode impedances were kept below 5 kΩ in most cases (at least below 10 kΩ). We digitized the electrical signals with a sampling rate of 500 Hz (Schaadt, Männel, van der Meer, Pannekamp, & Friederici, 2015).

### 2.5 EEG data processing and analysis

Recordings were algebraically re‐referenced to the average of both mastoids (A1, A2). To remove muscle artifacts from the EEG signal, a digital low‐pass filter of 30 Hz was applied to each single subject dataset (−3 dB cutoff frequency of 26.27 Hz). The sampling rate was then reduced to 250 Hz and a high‐pass filter of 0.5 Hz was applied to remove very slow drifts (−3 dB cutoff frequency of 0.501 Hz). Single EEG epochs or trials with a signal above ±80 μV within a sliding window of 200 ms were considered invalid (e.g., containing artifacts) and excluded.

The EEG data were averaged per participant and per condition (i.e., standards and deviants) between −200 and 1,250 ms relative to the onset of the stimuli. The response to the standard stimulus, which was presented directly after a deviant stimulus, was excluded from further analyses. A period of −200 to 0 ms relative to the stimulus onset was chosen for baseline correction. In a second step, grand averages were computed for each condition across subjects. All EEG processing was carried out with the EEP 3.2.1 software package (Max Planck Institute for Human Cognitive and Brain Sciences, Leipzig, Germany). Overall 20.49% of standard syllables (*SD* = 11.15; range: 0.48–46.90) and 19.81% of deviant syllables (*SD* = 12.44; range: 0.00–48.89) were excluded from further analyses. These numbers did not differ significantly between stimulus type (i.e., standard vs. deviant syllable; *p* = .47).

Individual MMR was quantified as the mean signal within 300–600 ms after stimulus onset. This represents the time window for which Schulte‐Körne et al. (1998) found significant differences between individuals with and without dyslexia in response to speech stimuli. All following analyses were computed on an anterior region of interest (ROI) (F3, Fz, F4), because of the typically found frontal distribution of the MMR (e.g. Näätänen, Paavilainen, Rinne, & Alho, 2007).

### 2.6 DNA extraction and genotyping

Saliva samples were used for genotyping and DNA extraction. DNA was extracted using standard procedures as described by Quinque, Kittler, Kayser, Stoneking, and Nasidze (2006) or using Oragene DNA Genotek Kits (Kanata, ON, Canada).

Genotyping was performed with the matrix‐assisted laser desorption/ionization time‐of‐flight mass spectrometry system iPLEX (Agena, Hamburg, Germany). Genotyping data had to fulfill the following quality measures: SNP‐wise Hardy‐Weinberg Equilibrium (HWE; *p* > .05 Bonferroni corrected), SNP‐wise call rate >97%, individual‐wise call rate >90%, and minor allele frequency (MAF) >0.05.

In total, 25 independent SNPs reported to be associated with dyslexia or dyslexia‐related phenotypes in at least two independent studies were investigated: rs16973771, rs2875891, and rs8053211 from *ATP2C2*; rs3935802, rs6564903, and rs7201632 from *CMIP*; rs10246256 and rs759178 from *CNTNAP2*; rs1419228, rs7765678, rs793862, and rs807701 from *DCDC2*; rs17819126, rs3743204, rs3743205, and rs685935 from *DYX1C1*; rs12533005 from *FOXP2*; rs2143340, rs2179515, rs6935076, rs761100, and rs9461045 from *KIAA0319*; rs1000585 from *MRPL19‐C2ORF3*; rs555879 from *MYO5B*; and rs12606138 from *NEDD4L*. In addition, two SNPs (rs11100040 and rs4234898) reported to be associated with the late component of MMR were included for analysis.

### 2.7 Statistical analyses

Differences in the averaged MMR signals among poor and good spelling probands were tested using a sliding window *t*‐test. In total, 375 time windows of 3.2 ms length were tested.

We used the literature driven 300–600 ms time window for association analysis in order to provide replicability among the different studies. Association analyses among genotyped SNPs and late MMR were conducted using a multifactorial linear regression model adjusted for poor spelling (categorized by the lower 10% percentile of the DERET outcome). Thereby, we used an additive genetic model. We analyzed the effect of ADD on the SNP‐MMR associations by comparing the effect sizes with and without adjusting for ADD. We verified that significant findings were not caused by influential outliers by performing regression applying Cook's distance. To analyze the distribution of the observed *p*‐values and to detect deviations from the expected *p*‐value distribution due to the multiple testing issue, a QQ‐plot was generated. The 95% confidence envelope is based on the order statistic of expected distribution. Thereby, we avoided bias due to linkage disequilibrium (LD) using only independent SNPs identified by clumping the SNP set by applying LD‐based clumping implemented in PLINK (Purcell et al., 2007) using standard settings. This clumping procedure identifies the subset of SNPs not strongly correlated, that is, being independent from each other thereby keeping stronger associated SNPs. The *p*‐values were controlled for multiple testing using the false discovery rate (FDR) (Benjamini & Hochberg, 1995).

An unweighted polygenic risk score (PRS) to estimate the joint discrimination capability was defined by summing up all risk alleles of the clumped, independent SNPs within each individual. This approach requires the information whether a certain allele can be considered a risk allele or not. We obtained this information from independent studies from the literature (Table S2) thereby avoiding bias as the independent studies from the literature serve as training sets. With this PRS, we analyzed the nexus between the late component of the MMR, dyslexia, and dyslexia candidate SNPs via a prediction approach. Therefore, we measured the prediction performance for poor spelling with the late component of MMR, the PRS and a combination of both components. As risk alleles were not reported for all candidate SNPs the risk score comprised of 20 of the 25 independent SNPs, no further filtering with regard to observed association levels was done. The prediction performance was assessed with the area under the ROC‐curve (AUC of ROC) for all three combinations. The AUC describes the ability of a model to discriminate between two groups (normal vs. poor spellers). Furthermore, prediction due to the addition of the PRS between good and poor readers was analyzed using different measures of reclassification (IDI and continuous NRI) applying the R add‐on package PredictABEL 1.2‐2 (Kundu, Aulchenko, & Janssens, 2014). Reclassification measures indicate if classification of cases and controls improves when adding new information (e.g., genetics) to the model (Müller et al., 2016).

All statistical analyses were performed using the R statistical software, Version 3.0.2 (R Core Team, 2013).

### 2.8 Power analyses

Our study had 80% power to detect an association of a SNP with the MMR at the level of nominal significance (*p*‐value = .05) for effect sizes of at least 13.1% explained variance in a linear regression model when accounting for spelling performance. At a level of *p*‐value .01 and .001 the power was 80% for effect sizes of 18.2% and 24.7% of explained variance, respectively. For calculating power, we used the framework of the general linear model as implemented in the R‐package pwr 1.1‐3 (Champely, 2015). The identified effect size corresponds to a medium to large effect size according to Cohen's classification (6%–16%, respectively). This is in accordance with previously reported effect sizes for the MMR phenotype (Roeske et al., 2011), which is until now—to the best of our knowledge—the only reported association of common genetic variants with dyslexia‐related MMR.

### 2.9 Functional in silico analyses

We characterized nominally associated SNPs, proxies of these SNPs (*R* ^2^ ≥ 0.3 and Lewontin's D′ ≥ 0.8), and respective genes for in silico evidence for functional effects. These investigations included eQTL analyses, annotations for local regulatory elements, and the investigation of the spatial distribution of genes and their expression products.

To identify eQTL effects, eQTL databases were analyzed (Borel et al., 2011; Dimas et al., 2009; Dixon et al., 2007; Fehrmann et al., 2011; Greenawalt et al., 2011; Grundberg et al., 2009; GTEx Consortium, 2015; Kim, Cho, Lee, & Webster, 2012; Kirsten et al., 2015; Mehta et al., 2013; Myers et al., 2007; Ramasamy et al., 2014; Schadt et al., 2008; Schröder et al., 2011; Veyrieras et al., 2008; Westra et al., 2013; Xia et al., 2012; Zeller et al., 2010). We only considered SNPs identified in brain or blood tissue and eQTLs had to be replicated in at least one study. We screened “RegulomeDB” (Boyle et al., 2012) for known and predicted local regulatory SNP functions. Tissue specificity of expressed proteins and RNA of respective genes was characterized using data from “The Human Protein Atlas” (Uhlen et al., 2015). Protein expression data from “The Human Protein Atlas” is derived from annotations of immune‐histochemical staining of various cell types across different tissues. RNA levels were determined by RNAseq experiments.

## 3 RESULTS

### 3.1 The late component of MMR discriminates between good and bad spellers

**Figure 1:**
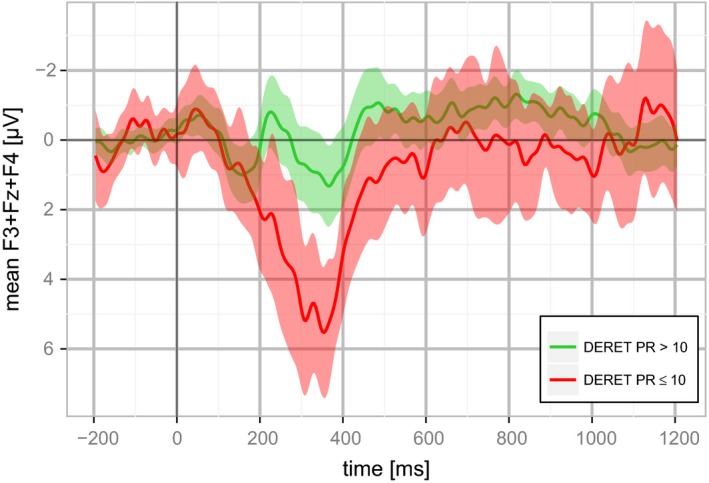
Difference wave (deviant‐standard) for the mean of F3, Fz, F4 stratified for spelling performance. Shaded regions correspond to the 95% confidence interval of the mean of the respective group according to *t*‐test statistics. Spelling performance was assessed using the DERET (Deutscher Rechtschreibtest; German Spelling Test)

Figure 1 shows averaged MMR signals of the frontal electrodes F3, Fz, and F4 from −200 ms before stimulus onset to 1,200 ms after stimulus onset. Children with normal spelling skills (DERET PR > 10%) presented a negativity at ~200 ms followed by a second negativity starting at ~400 ms after stimulus onset. In contrast, children with poor spelling skills (DERET PR ≤ 10%) exhibited a strong positivity of up to 5 μV between 200 and 600 ms. Differences among both groups were tested using a sliding window *t*‐test and revealed that MMR of both groups significantly differed within the interval between 200 and 400 ms (*p* < .05). This window is partially overlapping with the 300–600 ms window for which group‐wise differences between dyslexics and controls are often reported (Alonso‐Búa, Díaz, & Ferraces, 2006; Cheour, Korpilahti, Martynova, & Lang, 2001; Schulte‐Körne et al., 1998). Therefore, we decided to use the 300–600 ms time window (often called late component of the MMR) for all subsequent analyses in order to enhance comparability with previous studies. This late component of the MMR discriminated between good and poor spelling with an AUC of 0.78 (CI 95%: 0.67–0.89) in the presented study.

### 3.2 SNP selection, genotyping quality, and discrimination improvement

In total, 30 SNPs in 14 genes associated with dyslexia‐related phenotypes and replicated in at least one study were identified and investigated (Table S2). These SNPs corresponded to 25 independent SNPs. For 20 of those 25 independent SNPs, risk alleles were available in those studies previously published. In order to assess the relevance of these SNPs for dyslexia in our cohort, we investigated how a PRS created from those 25 SNPs discriminates between good and poor spelling individuals. When analyzing the PRS alone, we observed an AUC of 0.63 (CI 95% 0.497–0.78). When using the PRS in combination with the late component of MMR, we found an AUC increasing to 0.85 (CI 95%: 0.76–0.94). Thereby, the two measures ‘net reclassification improvement’ (NRI_cont_ = 0.72; *p* = .011) and ‘integrated discrimination improvement’ (IDI = 0.08; *p* = .019) revealed that an improved prediction was achieved by the PRS.

Finally, two additional SNPs (rs11100040 and rs4234898) were also selected for their previously reported association with the late component of the MMR in order to investigate whether this association can be replicated. All selected SNPs fulfilled the quality criteria (see Materials and Methods section).

### 3.3 Association of reported candidate SNPs with the late component of MMR

**Figure 2:**
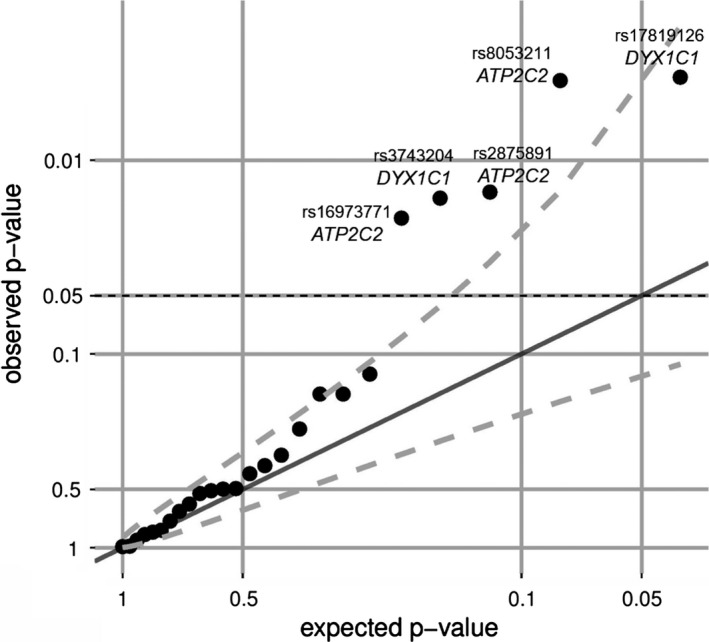
QQ‐plot of association analysis. The QQ‐plot displays the relation of the expected and the observed *p*‐value distribution for the 25 independent single‐nucleotide polymorphisms. The dashed line represents the 95% confidence interval revealing association stronger than expected due to multiple testing

In total, we identified five nominally associated SNPs at FDR of 11% representing independent genetic effects. SNP rs17819126‐*DYX1C1* (*p* = .0037) and *ATP2C2*‐rs8053211 (*p* = .0039) showed the strongest association at an FDR of 5%. Thereby, the SNP rs17819126‐*DYX1C1,* carriage of the allele previously reported for risk with the dyslexia‐related phenotype was associated with a more positive late component of the MMR. For the other four SNPs, carriage of the allele previously reported for risk with the dyslexia‐related phenotype was associated with decreased MMR levels (rs8053211, rs2875891, rs3743204, rs16973771, Table 2). We observed stronger association, that is, smaller *p*‐values than expected due to chance or multiple testing as shown in a QQ‐plot (Figure 2).

**Table 2:** Results of the association analysis

SNP	*p*‐Value	FDR	Beta	Gene
rs17819126	.0037	0.05	3.0	*DYX1C1*
rs8053211^a^	.0039	0.05	−1.8	*ATP2C2*
rs2875891^a^	.0146	0.11	−1.5	*ATP2C2*
rs3743204^a^	.0157	0.11	−1.7	*DYX1C1*
rs16973771^a^	.0199	0.11	−1.4	*ATP2C2*
rs11100040^b^	.0306	0.14	1.6	Intergenic

a Denotes SNPs with lower *p*‐values as expected from multiple testing. For details see the QQ‐plot shown in Figure 2.

Five independent SNPs previously reported to be associated with dyslexia or dyslexia‐related phenotypes in at least two studies revealed a nominal association with the late component of MMR. Furthermore, one SNP previously reported to be associated with MMR was successfully replicated in our study (b). The *p*‐values of the regression model are shown with the respective FDR. Effect size Beta corresponds to carriage of the previously reported risk allele in the literature.

Finally, when testing the two previously reported MMR‐related SNPs rs11100040 and rs4234898, we found a nominal significant association of rs11100040 with the late component of the MMR, where carriage of the previously reported risk alleles correlated with more positive MMR.

**Figure 3:**
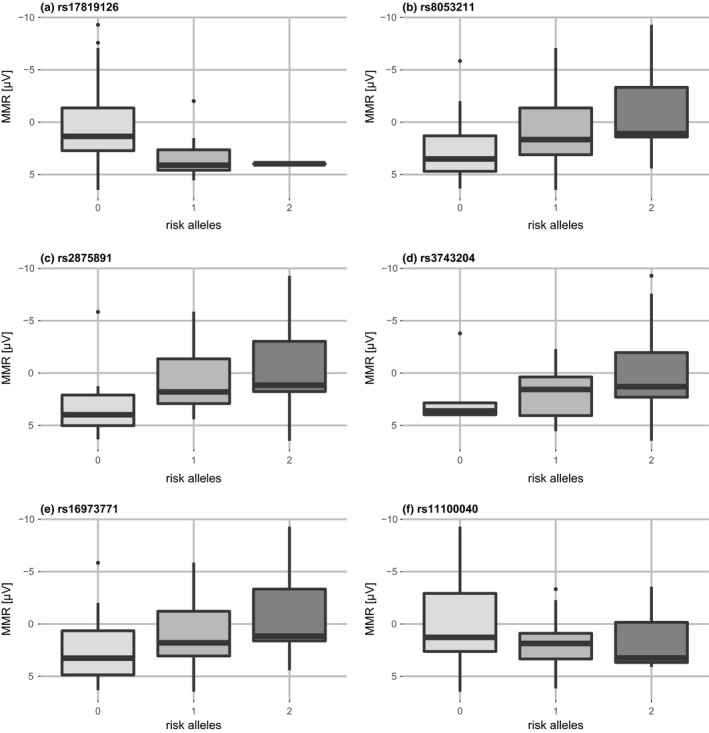
Effects of the nominal significant associated single‐nucleotide polymorphisms (SNPs) on MMR. Boxplots are shown according to the number of risk alleles of each SNP

Effect sizes of all nominally associated SNPs with the late component of the MMR are provided in Figure 3.

In order to investigate the relevance of ADD for our findings, we conducted an additional analysis where we adjusted for ADD status. Corroboratingly, we found only little change in *p*‐values and effect sizes when additionally adjusting our analysis on ADD status (Figure S1).

### 3.4 Functional characterization of identified candidate SNPs and corresponding genes

The screen for eQTL effects revealed direct *cis*‐effects for rs11100040, rs17819126, and rs3743204 in blood‐derived monocytes (Table S3), that is, for these SNPs, carriage of risk alleles is correlated with the expression level of certain genes. These genes were *PTPRU* (rs11100040), *PIGB* (rs17819126), and *DYX1C1* (rs3743204). When extending the search for proxy SNPs (*R* ^2^ ≥ 0.3 & D′ ≥ 0.8) to better identify possible correlating causative variants, additional evidence for a cis‐eQTL effect was found (*PIGB* for rs3743204 and *RAB27A* for rs17819126) in brain tissue.

The in silico characterization of associating SNPs using the “RegulomeDB” database identified evidence for transcription factor binding for SNP rs16973771. This was concluded on the basis of data from ChIP‐seq experiments, predictions on transcription factor binding sites, and evidence for open chromatin states from DNase‐seq, as well as footprinting experiments (Table S4). SNP rs3743204 showed evidence for protein binding from ChIP‐seq experiments and for open chromatin structures from DNase‐seq assays. Minimal binding evidence was reported for rs17819126 and rs8053211 with either evidence for protein binding from ChIP‐seq experiments or for open chromatin states from DNase‐seq experiments. No information was available for SNP rs11100040.

All genes were expressed in neuronal tissues and all were abundant as RNA in the cerebral cortex (Table S5).

## 4 DISCUSSION

Several studies show that an altered late component of the MMR, known to be connected to auditory discrimination, is associated with dyslexia (Hommet et al., 2009; Neuhoff et al., 2012; Schulte‐Körne et al., 1998). However, little is known about its genetic correlate. In this study, we identified links between a late component of the MMR and replicated candidate SNPs for dyslexia or dyslexia‐related phenotypes. First, we demonstrated validity of selected 25 independent SNPs for dyslexia in our sample by showing that additional inclusion of a PRS improved prediction of impaired writing. Secondly, we identified five independent SNPs out of 25 investigated SNPs (20%) to be associated with the late component MMR (*p* < .05) with four independent SNPs showing stronger association than expected due to multiple testing and two SNPs with an association at an FDR of 5%. Furthermore, one SNP (rs11100040) previously associated with the late component of MMR was also nominally replicated in our study. Thereby, we controlled for environmental influences on the MMR by analyzing the spelling‐independent MMR resulting from our adjustment strategy. Using functional data, we characterized these SNPs and corresponding genes.

### 4.1 Discrimination between good and poor spelling by MMR and genetics

In accordance with the literature (Lovio et al., 2010; Roeske et al., 2011; Schulte‐Körne et al., 1998, 2001), the late MMR significantly discriminated between people with good and poor spelling further corroborating its value as potential endophenotype for dyslexia. Also in accordance with previous reports, discrimination was found to be strong in a time window near 400 ms (Figure 1, Alonso‐Búa et al., 2006; Cheour et al., 2001). The validity of selected SNPs for dyslexia in our cohort was strengthened as we found significant improvement in reclassification good and poor spelling probands when using of a score created from these SNPs in addition to the late component of the MMR. To the best of our knowledge, this is the first report of a genetic risk score for dyslexia improving prediction.

### 4.2 Identified genetic modifiers of MMR

Next, we analyzed which subset of the selected SNPs might be associated with the late component of the MMR. We observed stronger associations than expected due to multiple testing with the late component of MMR (Figure 2), which supports a potential relationship between dyslexia and the late component of MMR on the genetic level. Thereby, the associations of these five SNPs represent a novel finding. However, results for a few other SNPs associating with MMR are available: Roeske et al. (2011) reported an association with the two‐marker haplotype rs4234898‐rs11100040 with the late component of the MMR in a set of 200 dyslexic children in a GWAS. Both SNPs of the haplotype were associated with altered expression levels of *SLC2A3*. This gene is a facilitated glucose transporter possibly involved in memory‐related processes as indicated by reduced GLUT3 (*SLC2A3*) levels in patients with Alzheimer's disease (Liu, Liu, Iqbal, Grundke‐Iqbal, & Gong, 2008). Here, we could also identify a nominal significant association of rs11100040 with the late component of the MMR. Interestingly, in a recent study, we were able to show that rs11100040 also affects the functional connectivity of the fronto‐temporal processing hubs in German native speakers (Skeide et al., 2015). The reduced functional connectivity between frontal and temporal brain areas might provide the basis for the dyslexia‐related modulation of the late component of the MMR because at least two regions are involved in generating the MMR: a frontal source is located in the inferior frontal gyrus and a temporal source located in the superior temporal gyrus (Doeller et al., 2003; Marco‐Pallarés, Grau, & Ruffini, 2005). Thus, the affected functional connectivity of the fronto‐temporal processing hubs, might lead to the observed functional alteration in the late component of the MMR for different numbers of risk alleles of rs11100040. Importantly, the study by Skeide et al. (2015) and the present analysis were conducted in overlapping cohorts, which further strengthen the proposed connection between the functional connectivity of the fronto‐temporal processing hubs, altered late component of the MMR and rs11100040.

In addition to Roeske et al. (2011), a second study investigated the association of genetic variants with the MMR (Czamara et al., 2011). However, this study was restricted to variants in *DCDC2* and *KIAA0319,* where one rare variant within *DCDC2* was associated with altered MMR. None of these rare genetic variants were investigated in our study since we included only common variants as we filtered for MAF ≥ 0.05. In accordance with Czamara et al. (2011), we also did not observe any association of common SNPs (MAFs ≥ 0.05) within *DCDC2* and *KIAA0319* with the late component of the MMR.

Our strongest identified associations include effects of SNPs in genes *DYX1C1* and *ATP2C2* on late component of the MMR (Table 2). The two strongest SNPs revealed associations with an FDR of 5%, which means that for both SNPs the probability of being a false discovery due to multiple testing is only 5%. Reflecting the candidate approach, all these genes are of high neurobiological relevance: *DYX1C1* (dyslexia susceptibility 1 candidate 1) encodes a protein expressed in cortical neurons and white matter glial cells (Taipale et al., 2003) and *DYX1C1* contributes to neuronal migration in the developing rodent brain (Wang et al., 2006). *ATP2C2* is an ATPase which transports Mg^2+^ and Ca^2+^ ions into the Golgi lumen for protein modification and is also involved in Ca^2+^ signaling (Feng et al., 2010). Interestingly, it is known that an imbalanced ion transmembrane gradient may impact neurological functions and supports the nexus between neurological impairment and risk for dyslexia.

It should be noted that our study was adequately powered to detect nominal associations for effect sizes similar to those described in Roeske et al. (2011) (see Materials and Methods). Therefore, we cannot exclude associations of other SNPs with the late component of the MMR at low effect sizes.

### 4.3 Characterization of the Effect Directions of Associated SNPs on the Late Component of the MMR

We observed a significant, positive MMR for children with poor spelling skills (Figure 1). This is in line with the reported stronger shift of the late component of the MMR toward positive values compared with children not being at risk for dyslexia by Maurer, Bucher, Brem, and Brandeis (2003) in kindergarteners at risk for dyslexia. Consequently, a stronger positivity of the late component of the MMR in relation to the number of risk alleles would be a straight‐forward assumption (Figure S2). However, we only observed the expected effect direction for the strongest associated SNP (Table 2). The inconsistent distribution of the effects of the other nominal significant SNPs on the late component of the MMR amplitude may be related to the well‐known phenomenon in genetics called “flip‐flop” association. Among others, it can be explained by differences in the underlying population structures where a causal variant in close proximity to the analyzed SNP arises from distinct founder mutations. These independent mutations manifest in divergent allele frequencies for the observed SNP in different populations. This in turn can lead to contradicting risk alleles in distinct populations for this SNP (Lin, Vance, Pericak‐Vance, & Martin, 2007). In fact, these “flip‐flop” associations are relatively common in dyslexia studies. For example, Taipale et al. (2003) identified an association of two SNPs (rs3743205‐*DYX1C1* and rs57809907‐*DYX1C1*) with dyslexia, thereby reporting −3A and T as risk alleles. Two subsequent studies replicated these findings for rs57809907, albeit with the opposite effect direction (Scerri et al., 2004; Wigg et al., 2004). Furthermore, several studies failed to replicate the initial association of rs3743205‐*DYX1C1* found by Taipale et al. (2003) (Bellini et al., 2005; Brkanac et al., 2007; Marino et al., 2005, 2007; Newbury et al., 2011; Saviour et al., 2008; Wigg et al., 2004).

Similarly, the identified effect sizes of SNP rs2143340‐*KIAA0319* were reported with opposing risk alleles (Francks et al., 2004; Luciano et al., 2007; Newbury et al., 2011). Contradicting effect size directions of risk alleles were also observed in studies investigating intermediate phenotypes of other diseases: Shulman et al. (2010) observed associations of *ZNF224* (rs3746319) and *PCK1* (rs8192708) with impaired cognition, an intermediate phenotype of Alzheimer's disease. The effect direction of these SNPs on the intermediate phenotype was opposite to the direction of the association with Alzheimer's disease seen in the initial GWAS. Here, the authors explained the “flip‐flop” association by differences in subject recruitment and ascertainment, with cross‐sectional versus prospective cohorts being a good example. This might also contribute to the differences in the effect direction we observed: for dyslexia, different cognitive subtypes are described (Heim et al., 2008; van Ermingen‐Marbach, Pape‐Neumann, Grande, Grabowska, & Heim, 2013) and different, subtype‐specific compositions of the case‐cohorts are plausible. Thus, if an allele is subtype‐specific, contradicting effect directions for a certain SNP are not unlikely.

### 4.4 Functional properties of associated SNPs

Most of the SNPs detected in association studies are intronic and do not change the protein structure. However, several studies have shown an effect of SNPs on gene expression levels. To follow this, we screened published eQTL databases for the significant SNPs or the best proxy of these SNPs. We regard functional evidence as an additional indicator for a true function of the respective SNP in dyslexia‐related phenotypes. Indeed, four associated SNPs could be linked to altered expression of nearby genes (*cis*‐eQTL, see Table S3). Note that with the exception of *DYX1C1* all these SNPs affect expression of other nearby genes to which they were not originally assigned to. Therefore, future research should consider these nearby genes as candidates when further investigating molecular pathomechanisms. We only considered eQTLs identified in brain tissue and blood‐derived cells because we expect that eQTLs involved in the development of dyslexia are likely present in brain tissue. However, we also included eQTL studies done in blood‐based tissue as these studies are traditionally very well powered. Furthermore, it is known that a large proportion of eQTLs are not tissue‐specific, especially *cis*‐acting eQTLs (Petretto et al., 2006; Van Nas et al., 2010). Note that all genes affected by the associated eQTL were expressed in brain tissue (Uhlen et al., 2015) which strengthens the proposed functional effect of these genes on dyslexia. As the number of eQTL studies increases, we expect even more findings in the future including relevant eQTLs in cerebral tissue.

We further examined the analyzed SNPs for functional in silico evidence to obtain possible molecular mechanisms for functional effects. For most of the analyzed SNPs, in silico functional data on the genetic level was found (Boyle et al., 2012), for example, footprinting and position weight matrix assays (PWM) provided evidence for rs16973771‐*ATP2C2* to affect the binding of Nuclear factor 1 (NF‐1) transcription factor family members (Matys et al., 2006; Pique‐Regi et al., 2011). A complete list is shown in Table S4.

### 4.5 Limitations

We would like to address some limitations regarding this study. Due to our moderate sample size our results should be followed up in independent replication studies and when interpreting effect sizes the well‐known winner's curse (Lohmueller, Pearce, Pike, Lander, & Hirschhorn, 2003) should be taken into consideration. Based on the findings of this study, we cannot make definite conclusions about the role of the analyzed genetic variants. Nevertheless, our results are supported by in silico functional data and a previous genetic MMR study (Roeske et al. (2011) and are available for meta‐analysis in future studies.

As another potential limitation, our study sample was a homogenous Caucasian group, therefore, our results might differ in other ethnicities.

## 5 CONCLUSION

This study provides further evidence for genetic variants within *DYX1C1* and *ATP2C2* as candidates for dyslexia. For these SNPs, our study suggests a pathomechanistical link with the late component of the MMR possibly via modulating gene expression regulation. However, these findings should be further investigated in additional samples. Moreover, our results corroborate the late component of MMR as a potential neurophysiological endophenotype for dyslexia and show that dyslexia candidate SNPs can improve the predictive power of the late component of the MMR. Validation of candidate SNPs and characterization of their functional effects may be helpful for the development of diagnostic tools and the ongoing understanding of the molecular pathomechanisms of dyslexia.

## CONFLICT OF INTEREST

All contributing authors are members of the Legascreen consortium. The aim of this consortium is to develop an early screening test for dyslexia based on biological markers. Arndt Wilcke, Johannes Boltze, Frank Emmrich, and Holger Kirsten hold a patent concerning the genetic diagnosis of dyslexia.
