# Supplementary material for: ATP2C2 and DYX1C1 are putative modulators of dyslexia‐related MMR
Source: Brain Behav. 2017 Oct 18;7(11):e00851. doi: 10.1002/brb3.851 (PMC5698869; doi:10.1002/brb3.851)
Supplement: Supplementary file 1 [file BRB3-7-e00851-s001.pdf]

Supporting Information

ATP2C2 and *DYX1C1* are putative modulators of dyslexia-related MMR

Bent Müller, Gesa Schaadt, Johannes Boltze, Frank Emmrich, LEGASCREEN consortium, Michael A. Skeide, Nicole E. Neef, Indra Kraft, Jens Brauer, Angela D. Friederici, Holger Kirsten, Arndt Wilcke

Supporting Information 1. DERET description

The text for third and fourth graders consists of ten sentences. Before writing the text from dictation, the text is completely read to the children once. When dictation begins, the first sentence is dictatated completely and after, segments (i.e., each consisting of three words on average) of the sentence are dictated to the child sequentially. After the segment of the sentence has been written down the next segment of the sentence is dictated. This procedure is continued for each of the following nine sentences. Spelling mistakes are enumerated for each sentence (one mistake is noted for words with at least one spelling mistake), summed up for the whole text, and translated into age-normed percentile ranks (PR).

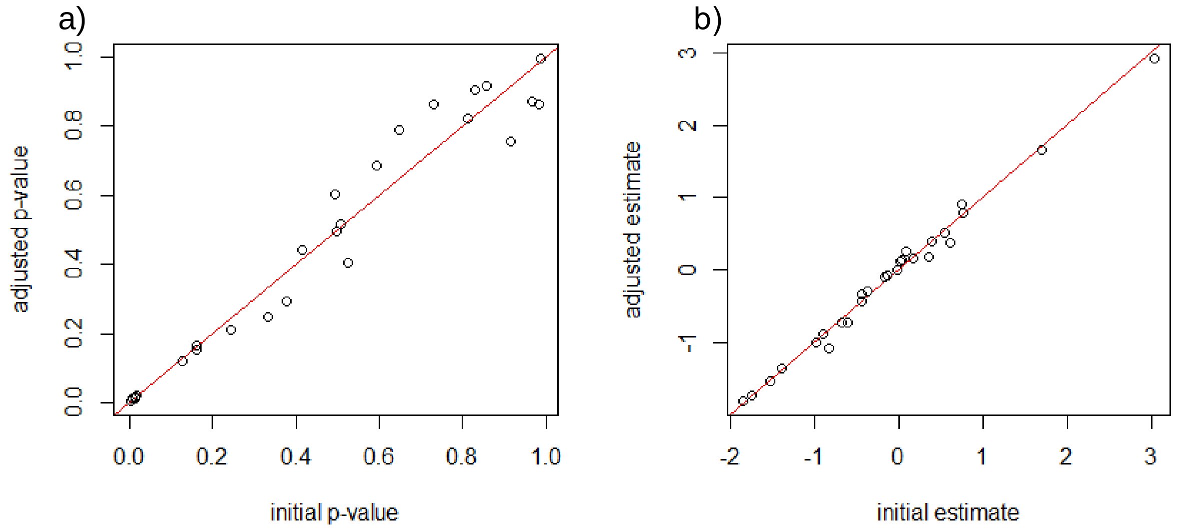

Supporting Figure 1. Correlation between the ADD-unadjusted and ADD-adjusted p-values (a) and ADD-unadjusted and ADD-adjusted effect sizes (b).
Here, values of the ADD-unadjusted model are represented on the x-axes, whereas values of the ADD-adjusted model are shown on the y-axes.
The ADD-unadjusted model is specified as associations among SNPs and the late component of the MMR adjusted for poor spelling as used in the main analysis. The ADD-unadjusted model is the same but additionally includes ADD status as covariate.

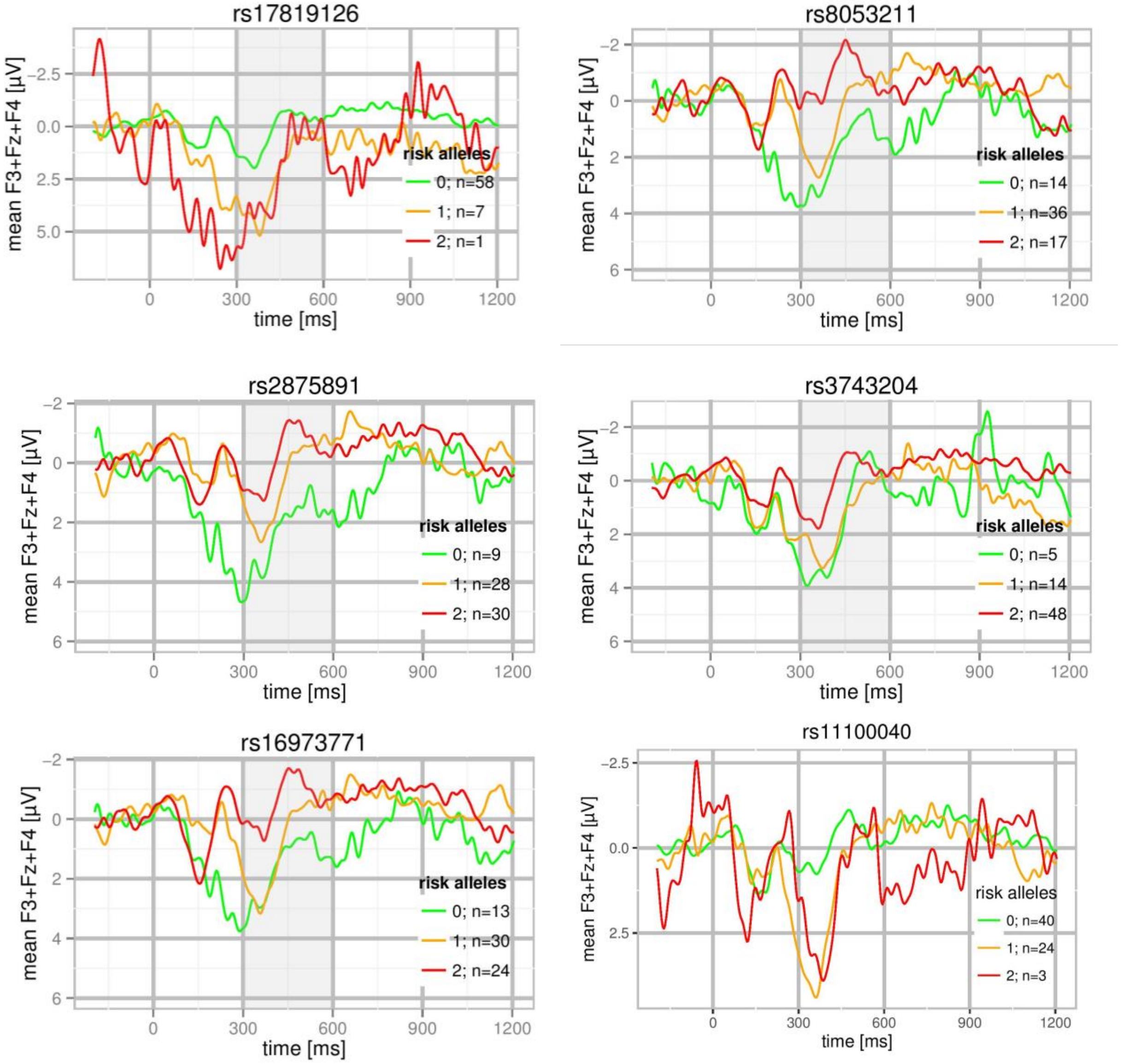

Supporting Figure 2. Difference waves (deviant-standard) for the mean of F3, Fz, F4 stratifed according to the number of risk alleles for each of the nominal significant associated SNPs.

Supporting Table 1. Details of the spelling subgroups. The p-values give the defference between the DERET > 10 and DERET≤ 10 groups in the respective measures.

	DERET > 10	DERET ≤ 10	p-value
Boys : girls	28:25	10:4	0.243
Age	9.65 ± 0.53	9.54 ± 0.49	0.485
ADD	6	6	0.013
Nonverbal intelligence (mean IQ)	112 ± 9.7	106 ± 6.5	0.013

Supporting Table 2. Overview of analyzed SNPs. Presented are the SNPs with the respective gene, the minor allele frequency (MAF) of the genotyped SNP, the independent, tagging lead-SNP idenitifed by clumping, the p-value, the estimate of the main effect and the risk allele of the respective SNP. SNPs were annotated with genes they were located in or if not applicable the nearest gene with the distance to the SNP in parentheses. SNP / gene informations are based on GRCh37.

SNP	Gene	MAF	Lead SNP	p-value	Estimate	References	Coded as risk allele
rs17819126	*DYX1C1*	0.07		0.0037	3	Bates *et al.*, 2010; Paracchini *et al.*, 2011	minor
rs8053211	*ATP2C2*	0.48		0.0039	-1.8	Newbury *et al.*, 2009, 2011; Scerri *et al.*, 2011	major
rs11860694	*ATP2C2*	0.48	rs8053211	0.0096	1.7	Newbury *et al.*, 2009, 2011	minor
rs16973771	*ATP2C2*	0.42		0.0199	-1.4	Newbury *et al.*, 2009, 2011	major
rs2875891	*ATP2C2*	0.34		0.0146	-1.5	Newbury *et al.*, 2009, 2011	major
rs3743204	*DYX1C1*	0.17		0.0157	-1.7	Bates *et al.*, 2010; Becker *et al.*, 2013; Dahdouh *et al.*, 2009; Wigg *et al.*, 2004	major
rs8045507	ATP2C2	0.42	rs16973771	0.0199	-1.4	Newbury *et al.*, 2009, 2011	major
rs10246256	*CNTNAP2*	0.3		0.1606	-0.9	Newbury *et al.*, 2011; Vernes *et al.*, 2008	major
rs12533005	*FOXP2*	0.39		0.1268	-1	Peter *et al.*, 2011; Wilcke *et al.*, 2012	major
rs6564903	*CMIP*	0.41		0.1612	0.8	Newbury *et al.*, 2009, 2011; Scerri *et al.*, 2011	major
rs3935802	*CMIP*	0.38		0.2439	-0.7	Newbury *et al.*, 2009, 2011	minor
rs9461045	*TDP2* (1.1kb)	0.2		0.3333	0.8	Dennis *et al.*, 2009; Newbury *et al.*, 2011	minor
rs3212236	*TDP2* (1.7kb)	0.2	rs9461045	0.3333	0.8	Harold *et al.*, 2006; Newbury *et al.*, 2011	minor
rs6935076	*KIAA0319*	0.38		0.3766	-0.6	Becker *et al.*, 2013; Cope *et al.*, 2005; Couto *et al.*, 2010; Luciano *et al.*, 2007; Newbury *et al.*, 2011; Paracchini *et al.*, 2008; Scerri *et al.*, 2011	minor
rs555879	*MYO5B*	0.45		0.415	0.5	Scerri *et al.*, 2010	minor
rs685935	*DYX1C1*	0.41		0.4949	-0.4	Bates *et al.*, 2010; Paracchini *et al.*, 2011	minor
rs759178	*CNTNAP2*	0.48		0.498	-0.4	Vernes *et al.*, 2008; Whitehouse *et al.*, 2011	major
rs2710102	*CNTNAP2*	0.48	rs759178	0.498	-0.4	Newbury *et al.*, 2011; Peter *et al.*, 2011; Vernes *et al.*, 2008; Whitehouse, Bishop, Ang, Pennell, & Fisher, 2011	major
rs7201632	*CMIP*	0.43		0.508	0.4	Newbury *et al.*, 2009, 2011	major
rs3743205	*DYX1C1*	0.43		0.5253	-0.8	Becker *et al.*, 2013;	major

SNP	Gene	MAF	Lead SNP	p-value	Estimate	References	Coded as risk allele
						Dahdouh *et al.*, 2009;<br>Lim, Ho, Chou, & Waye, 2011;<br>Newbury *et al.*, 2011;<br>Taipale *et al.*, 2003;<br>Wigg *et al.*, 2004	
rs793862	*DCDC2*	0.3		0.5947	-0.4	Becker *et al.*, 2013;<br>Ludwig *et al.*, 2008;<br>Meng *et al.*, 2005;<br>Scerri *et al.*, 2011;<br>Schumacher *et al.*, 2006; Wilcke *et al.*, 2009	minor
rs7765678	*DCDC2*	0.08		0.6492	0.6	Lind *et al.*, 2010	major
rs12606138	*NEDD4L*	0.4		0.7294	0.4	Scerri *et al.*, 2010	major
rs2179515	*KIAA0319*	0.33		0.8117	0.2	Becker *et al.*, 2013;<br>Cope *et al.*, 2005	major
rs807701	*DCDC2*	0.4		0.8311	-0.1	Becker *et al.*, 2013;<br>Ludwig *et al.*, 2008;<br>Schumacher *et al.*, 2006;<br>Wilcke *et al.*, 2009	minor
rs1419228	*DCDC2*	0.19		0.8552	-0.2	Lind *et al.*, 2010;<br>Paracchini *et al.*, 2011	minor
rs2143340	*KIAA0319*	0.19		0.9142	0.1	Francks *et al.*, 2004;<br>Luciano *et al.*, 2007;<br>Newbury *et al.*, 2011;<br>Paracchini *et al.*, 2008, 2011	minor
rs8094327	*NEDD4L*	0.13	rs12606138	0.9665	0	Scerri *et al.*, 2010	minor
rs1000585	*EVA1A* (26kb)	0.41		0.982	0	Anthoni *et al.*, 2007;<br>Newbury *et al.*, 2011	minor
rs761100	*KIAA0319*	0.41		0.9874	0	Becker *et al.*, 2013;<br>Harold *et al.*, 2006;<br>Ludwig *et al.*, 2008;<br>Newbury *et al.*, 2011	minor
rs11100040	*FTH1P21 (79kb)*	0.23		0.0306	1.6	Roeske *et al.*, 2011	
rs4234898	*FTH1P21 (70kb)*	0.14		0.1711	1.2	Roeske *et al.*, 2011	

Supporting Table 3. Literature-screen for eQTL-effects. Displayed are the SNP with their respective gene, the linkage (R^2) to the eQTL, the gene which expression is affected by the eQTL, the tissue the effect was observed and the respective study. If the eQTL and the tested SNP are identical, the eQTL is shown in bold.

**SNP**	**Gene**	**eQTL**	**R^2**	**D'**	**Affected gene**	**Tissue**	**Study**
rs3743204	*DYX1C1*	**rs3743204**	1.00	1.00	*DYX1C1*	Blood	Heinzen *et al.,* 2008
rs3743204	*DYX1C1*	chr15_55765580_I	0.31	0.82	*DYX1C1-CCPG1*	Brain (Cerebellum)	GTEx Consortium,, 2015
rs3743204	*DYX1C1*	rs8040756	0.57	0.90	*CCPG1*	Blood	Fehrmann *et al.,* 2011
rs3743204	*DYX1C1*	rs8040756	0.57	0.90	*PIGB*	Blood	Fehrmann *et al.,* 2011
rs3743204	*DYX1C1*	rs8037056	0.44	0.98	*PIGB*	Monocytes	Zeller *et al.,* 2010
rs17819126	*DYX1C1*	**rs17819126**	1.00	1	*PIGB*	Blood	Westra *et al.*, 2013
rs17819126	*DYX1C1*	rs4774760	0.30	1	*PIGB*	Monocytes	Zeller *et al.,* 2010
rs17819126	*DYX1C1*	rs11857829	0.95	1	*RAB27A*	Blood	Fehrmann *et al.,* 2011
rs17819126	*DYX1C1*	rs11855084	0.95	1	*RAB27A*	Blood	Westra *et al.*, 2013
rs11100040	*FTH1P21* (79kb)	**rs11100040**	1.00	1	*PTPRU*	Blood	Fehrmann *et al.,* 2011
rs11100040	*FTH1P21* (79kb)	**rs11100040**	1.00	1	*PTPRU*	Blood	Westra *et al.*, 2013

Supporting Table 4. Known and predicted regulatory elements for all associated SNPs. Displayed is the full output of the RegulomeDB.

SNP	Hits
**rs16973771**	Motifs|Footprinting|8988t|NF-1, Motifs|PWM||NF-1, Chromatin_Structure||DNase-seq|8988t, Chromatin_Structure||DNase-seq|Ips, Chromatin_Structure|Diffa14d|DNase-seq|H7es, Chromatin_Structure||DNase-seq|K562G1phase, Chromatin_Structure||DNase-seq|Lncap, Chromatin_Structure|Nabut|DNase-seq|K562, Chromatin_Structure||DNase-seq|K562, Chromatin_Structure|Saha1u72hr|DNase-seq|K562, Chromatin_Structure||DNase-seq|H7es, Protein_Binding||ChIP-seq|K562|EZH2
**rs3743204**	Chromatin_Structure|Lenticon|DNase-seq|Fibroblgm03348, Chromatin_Structure||DNase-seq|Adultcd4th0, Chromatin_Structure|Ifna4h|FAIRE|Helas3, Chromatin_Structure||DNase-seq|Fibrobl, Chromatin_Structure||DNase-seq|Ipsnihi7, Chromatin_Structure||DNase-seq|Htr8, Chromatin_Structure||DNase-seq|K562, Chromatin_Structure|Tam10030|DNase-seq|Ishikawa, Chromatin_Structure||DNase-seq|Fibropag08395, Chromatin_Structure|Andro|DNase-seq|Lncap, Chromatin_Structure||DNase-seq|Gcbcell, Chromatin_Structure||DNase-seq|Hpde6e6e7, Chromatin_Structure||DNase-seq|Mel2183, Chromatin_Structure||DNase-seq|Cll, Chromatin_Structure||FAIRE|Helas3, Chromatin_Structure|Est10nm30m|DNase-seq|Ishikawa, Chromatin_Structure||DNase-seq|Cd20ro01794, Chromatin_Structure||DNase-seq|Ipsnihi11, Chromatin_Structure||DNase-seq|Ips, Chromatin_Structure||DNase-seq|Mcf7, Chromatin_Structure||DNase-seq|Heartoc, Chromatin_Structure|Ifng4h|FAIRE|Helas3, Chromatin_Structure||DNase-seq|Fibrop, Chromatin_Structure||DNase-seq|UrotsaUt189, Chromatin_Structure||DNase-seq|Panisd, Chromatin_Structure||DNase-seq|Fibroblgm03348, Chromatin_Structure||DNase-seq|Cerebellumoc, Chromatin_Structure||DNase-seq|Gm10266, Chromatin_Structure||DNase-seq|Nhek, Chromatin_Structure||DNase-seq|Sknsh, Chromatin_Structure|Hypoxlaccon|DNase-seq|Mcf7, Chromatin_Structure||DNase-seq|T47d, Chromatin_Structure||DNase-seq|Gliobla, Chromatin_Structure||DNase-seq|A549, Chromatin_Structure||DNase-seq|Huh7, Chromatin_Structure||DNase-seq|Lncap, Chromatin_Structure||DNase-seq|Helas3, Chromatin_Structure||DNase-seq|Gm19238, Chromatin_Structure||DNase-seq|Cerebrumfrontaloc, Chromatin_Structure||DNase-seq|Hbvsmc, Chromatin_Structure|Ifna4h|DNase-seq|Helas3, Chromatin_Structure||FAIRE|Nhek, Chromatin_Structure||DNase-seq|Hek293t, Chromatin_Structure||DNase-seq|Urotsa, Chromatin_Structure|Est10nm30m|DNase-seq|T47d, Chromatin_Structure||DNase-seq|Gm12891, Chromatin_Structure||DNase-seq|Rwpe1, Chromatin_Structure||DNase-seq|Gm19239, Chromatin_Structure||DNase-seq|Huh75, Chromatin_Structure||DNase-seq|Gm19240, Chromatin_Structure||DNase-seq|Osteobl, Chromatin_Structure|Est10nm30m|DNase-seq|Ecc1, Chromatin_Structure||DNase-seq|Phte, Chromatin_Structure||DNase-seq|Naivebcell, Chromatin_Structure||DNase-seq|Olfneurosphere, Chromatin_Structure||DNase-seq|Stellate, Chromatin_Structure||DNase-seq|Gm13977, Chromatin_Structure||DNase-seq|Imr90, Chromatin_Structure|Lentimyod|DNase-seq|Fibroblgm03348, Chromatin_Structure||DNase-seq|Gm10248, Chromatin_Structure||DNase-seq|Hepg2, Chromatin_Structure|Hypoxlac|DNase-seq|Mcf7, Chromatin_Structure||DNase-seq|Ipscwru1, Chromatin_Structure||DNase-seq|Hsmmfshd, Chromatin_Structure||DNase-seq|Hsmmemb, Chromatin_Structure||DNase-seq|Fibropag20443, Chromatin_Structure||DNase-seq|Medullod341, Chromatin_Structure||DNase-seq|Medullo, Chromatin_Structure|Serumfree|DNase-seq|Aosmc, Chromatin_Structure||DNase-seq|Gm20000, Chromatin_Structure|Dm002p1h|DNase-seq|Ecc1, Chromatin_Structure||DNase-seq|Gm12892, Chromatin_Structure||DNase-seq|Hsmm, Chromatin_Structure||DNase-seq|Fibropag08396, Chromatin_Structure||DNase-seq|Gm12878, Chromatin_Structure||DNase-seq|Monocd14, Chromatin_Structure|Znf4g7d3|DNase-seq|K562, Chromatin_Structure||DNase-seq|Panislets, Chromatin_Structure||DNase-seq|Gm13976, Protein_Binding|shLuc|ChIP-seq|CD36|GATA1, Protein_Binding|02pct|ChIP-seq|A549|REST, Protein_Binding||ChIP-seq|GM12878|SIN3A, Protein_Binding|shbrg1|ChIP-seq|CD36|GATA1, Protein_Binding||ChIP-seq|PFSK-1|SIN3A, Protein_Binding||ChIP-seq|HeLa-S3|SMARCC1, Protein_Binding|tnfa|ChIP-seq|GM15510|NFKB1, Protein_Binding||ChIP-seq|K562|SAP30, Protein_Binding|02pct|ChIP-seq|A549|SIN3A, Protein_Binding||ChIP-seq|K562|RFX3, Protein_Binding||ChIP-seq|HeLa-S3|CEBPB
**rs17819126**	Chromatin_Structure||DNase-seq|Monocd14ro1746, Chromatin_Structure||DNase-seq|Monocd14
**rs8053211**	Chromatin_Structure||DNase-seq|K562, Chromatin_Structure|Saha1u72hr|DNase-seq|K562, Chromatin_Structure||DNase-seq|Mcf7, Chromatin_Structure|Nabut|DNase-seq|K562, Chromatin_Structure||DNase-seq|Hmec
**rs2875891**	Chromatin_Structure||DNase-seq|Monocd14ro1746, Chromatin_Structure||DNase-seq|Monocd14
**rs11100040**	No data

Supplementary Table 5. Expression characteristics of the associated genes. Expression levels according to protein detection and RNA detection are displayed. Protein expression was stratified according to tissue and cell type. RNA expression levels were categorized into low (1-10 FPKM), medium (10-50 FPKM) or high (>50 FPKM) RNA abundance.

**Region**	***DYX1C1***	***ATP2C2***
Protein level		
cerebellum / cells in granular layer	not detected	not detected
cerebellum / cells in molecular layer	medium	not detected
cerebellum / Purkinje cells	medium	not detected
cerebral cortex / endothelial cells	low	medium
cerebral cortex / glial cells	low	not detected
cerebral cortex / neuronal cells	medium	low
cerebral cortex / neuropil	not detected	not detected
hippocampus / glial cells	not detected	not detected
hippocampus / neuronal cells	medium	not detected
lateral ventricle / glial cells	medium	not detected
lateral ventricle / neuronal cells	medium	medium
RNA level		
cerebral cortex	low	low

References

Anthoni, H., Zucchelli, M., Matsson, H., Müller-Myhsok, B., Fransson, I., Schumacher, J., Massinen, S., Onkamo, P., Warnke, A., Griesemann, H., *et al.* (2007) A locus on 2p12 containing the co-regulated MRPL19 and C2ORF3 genes is associated to dyslexia. *Hum Mol Genet* **16**, 667–677.

Bates, T.C., Lind, P.A., Luciano, M., Montgomery, G.W., Martin, N.G., & Wright, M.J. (2010) Dyslexia and DYX1C1: deficits in reading and spelling associated with a missense mutation. *Mol Psychiatry* **15**, 1190–1196.

Becker, J., Czamara, D., Scerri, T.S., Ramus, F., Csépe, V., Talcott, J.B., Stein, J., Morris, A., Ludwig, K.U., Hoffmann, P., *et al.* (2013) Genetic analysis of dyslexia candidate genes in the European cross-linguistic NeuroDys cohort. *Eur J Hum Genet* 1–6.

Cope, N., Harold, D., Hill, G., Moskvina, V., Stevenson, J., Holmans, P., Owen, M.J., O'Donovan, M.C., & Williams, J. (2005) Strong evidence that KIAA0319 on chromosome 6p is a susceptibility gene for developmental dyslexia. *Am J Hum Genet* **76**, 581–591.

Couto, J.M., Livne-Bar, I., Huang, K., Xu, Z., Cate-Carter, T., Feng, Y., Wigg, K., Humphries, T., Tannock, R., Kerr, E.N., *et al.* (2010) Association of reading disabilities with regions marked by acetylated H3 histones in KIAA0319. *Am J Med Genet B Neuropsychiatr Genet* **153B**, 447–462.

Dahdouh, F., Anthoni, H., Tapia-Páez, I., Peyrard-Janvid, M., Schulte-Körne, G., Warnke, A., Remschmidt, H., Ziegler, A., Kere, J., Müller-Myhsok, B., *et al.* (2009) Further evidence for DYX1C1 as a susceptibility factor for dyslexia. *Psychiatr Genet* **19**, 59–63.

Dennis, M.Y., Paracchini, S., Scerri, T.S., Prokunina-Olsson, L., Knight, J.C., Wade-Martins, R., Coggill, P., Beck, S., Green, E.D., & Monaco, A.P. (2009) A common variant associated with dyslexia reduces expression of the KIAA0319 gene. *PLoS Genet* **5**, e1000436.

Fehrmann, R.S.N., Jansen, R.C., Veldink, J.H., Westra, H.-J.J., Arends, D., Bonder, M.J., Fu, J., Deelen, P., Groen, H.J.M., Smolonska, A., *et al.* (2011). Trans-eQTLs reveal that independent genetic variants associated with a complex phenotype converge on intermediate genes, with a major role for the HLA. *PLoS Genet* **7**, e1002197.

Francks, C., Paracchini, S., Smith, S.D., Richardson, A.J., Scerri, T.S., Cardon, L.R., Marlow, A.J., MacPhie, I.L., Walter, J., Pennington, B.F., *et al.* (2004) A 77-kilobase region of chromosome 6p22.2 is associated with dyslexia in families from the United Kingdom and from the United States. *Am J Hum Genet* **75**, 1046–1058.

GTEx Consortium (2015). Human genomics. The Genotype-Tissue Expression (GTEx) pilot analysis: multitissue gene regulation in humans. *Science* **348**, 648–660.

Harlaar, N., Butcher, L.M., Meaburn, E., Sham, P., Craig, I.W., & Plomin, R. (2005) A behavioural genomic analysis of DNA markers associated with general cognitive ability in 7-year-olds. *J Child Psychol Psychiatry* **46**, 1097–1107.

Harold, D., Paracchini, S., Scerri, T., Dennis, M., Cope, N., Hill, G., Moskvina, V., Walter, J., Richardson, a J., Owen, M.J., *et al.* (2006) Further evidence that the KIAA0319 gene confers susceptibility to developmental dyslexia. *Mol Psychiatry* **11**, 1085–1091, 1061.

Heinzen, E.L., Ge, D., Cronin, K.D., Maia, J.M., Shianna, K. V, Gabriel, W.N., Welsh-Bohmer, K.A., Hulette, C.M., Denny, T.N., & Goldstein, D.B. (2008). Tissue-Specific Genetic Control of Splicing: Implications for the Study of Complex Traits. *PLoS Biol* **6**, e1000001.

Lim, C.K.P., Ho, C.S.H., Chou, C.H.N., & Waye, M.M.Y. (2011) Association of the rs3743205 variant of DYX1C1 with dyslexia in Chinese children. *Behav Brain Funct* **7**, 16.

Lind, P.A., Luciano, M., Wright, M.J., Montgomery, G.W., Martin, N.G., & Bates, T.C. (2010) Dyslexia and DCDC2: normal variation in reading and spelling is associated with DCDC2 polymorphisms in an Australian population sample. *Eur J Hum Genet* **18**, 668–673.

Luciano, M., Lind, P.A., Duffy, D.L., Castles, A., Wright, M.J., Montgomery, G.W., Martin, N.G., & Bates, T.C. (2007) A haplotype spanning KIAA0319 and TTRAP is associated with normal variation in reading and spelling ability. *Biol Psychiatry* **62**, 811–817.

Ludwig, K.U., Roeske, D., Schumacher, J., Schulte-Körne, G., König, I.R., Warnke, A., Plume, E., Ziegler, A., Remschmidt, H., Müller-Myhsok, B., *et al.* (2008) Investigation of interaction between DCDC2 and KIAA0319 in a large German dyslexia sample. *J Neural Transm* **115**, 1587–1589.

Meng, H., Smith, S.D., Hager, K., Held, M., Liu, J., Olson, R.K., Pennington, B.F., DeFries, J.C., Gelernter, J., O'Reilly-Pol, T., *et al.* (2005) DCDC2 is associated with reading disability and modulates neuronal development in the brain. *Proc Natl Acad Sci U S A* **102**, 17053–17058.

Mueller, B., Ahnert, P., Burkhardt, J., Brauer, J., Czepezauer, I., Quente, E., Boltze, J., Wilcke, A., Kirsten, H., 2014. Genetic risk variants for dyslexia on chromosome 18 in a German cohort. *Genes Brain Behav* **13**, 350–6.

Newbury, D.F., Winchester, L., Addis, L., Paracchini, S., Buckingham, L.-L., Clark, A., Cohen, W., Cowie, H., Dworzynski, K., Everitt, A., *et al.* (2009) CMIP and ATP2C2 modulate phonological short-term memory in language impairment. *Am J Hum Genet* **85**, 264–272.

Newbury, D.F., Paracchini, S., Scerri, T.S., Winchester, L., Addis, L., Richardson, A.J., Walter, J., Stein, J.F., Talcott, J.B., & Monaco, A.P. (2011) Investigation of dyslexia and SLI risk variants in reading- and language-impaired subjects. *Behav Genet* **41**, 90–104.

Paracchini, S., Steer, C.D., Buckingham, L.-L., Morris, A.P., Ring, S., Scerri, T., Stein, J., Pembrey, M.E., Ragoussis, J., Golding, J., *et al.* (2008) Association of the KIAA0319 dyslexia susceptibility gene with reading skills in the general population. *Am J Psychiatry* **165**, 1576–1584.

Paracchini, S., Ang, Q.W., Stanley, F.J., Monaco, a P., Pennell, C.E., & Whitehouse, a J.O. (2011) Analysis of dyslexia candidate genes in the Raine cohort representing the general Australian population. *Genes Brain Behav* **10**, 158–165.

Peter, B., Raskind, W.H., Matsushita, M., Lisowski, M., Vu, T., Berninger, V.W., Wijsman, E.M., & Brkanac, Z. (2011) Replication of CNTNAP2 association with nonword repetition and support for FOXP2 association with timed reading and motor activities in a dyslexia family sample. *J Neurodev Disord* **3**, 39–49.

Roeske, D., Ludwig, K.U., Neuhoff, N., Becker, J., Bartling, J., Bruder, J., Brockschmidt, F.F., Warnke, A., Remschmidt, H., Hoffmann, P., *et al.* (2011) First genome-wide association scan on neurophysiological endophenotypes points to trans-regulation effects on SLC2A3 in dyslexic children. *Mol Psychiatry* **16**, 97–107.

Scerri, T.S., Paracchini, S., Morris, A., MacPhie, I.L., Talcott, J., Stein, J., Smith, S.D., Pennington, B.F., Olson, R.K., DeFries, J.C., *et al.* (2010) Identification of candidate genes for dyslexia susceptibility on chromosome 18. *PLoS One* **5**, e13712.

Scerri, T.S., Morris, A.P., Buckingham, L.L., Newbury, D.F., Miller, L.L., Monaco, A.P., Bishop, D.V.M., & Paracchini, S. (2011) DCDC2, KIAA0319 and CMIP are associated with reading-related traits. *Biol Psychiatry* **70**, 237–245.

Schumacher, J., Anthoni, H., Dahdouh, F., König, I.R., Hillmer, A.M., Kluck, N., Manthey, M., Plume, E., Warnke, A., Remschmidt, H., *et al.* (2006) Strong genetic evidence of DCDC2 as a susceptibility gene for dyslexia. *Am J Hum Genet* **78**, 52–62.

Taipale, M., Kaminen, N., Nopola-Hemmi, J., Haltia, T., Myllyluoma, B., Lyytinen, H., Muller, K., Kaaranen, M., Lindsberg, P.J., Hannula-Jouppi, K., *et al.* (2003) A candidate gene for developmental dyslexia encodes a nuclear tetratricopeptide repeat domain protein dynamically regulated in brain. *Proc Natl Acad Sci U S A* **100**, 11553–11558.

Vernes, S.C., Newbury, D.F., Abrahams, B.S., Winchester, L., Nicod, J., Groszer, M., Alarcón, M., Oliver, P.L., Davies, K.E., Geschwind, D.H., *et al.* (2008) A functional genetic link between distinct developmental language disorders. *N Engl J Med* **359**, 2337–2345.

Westra, H.-J., Peters, M.J., Esko, T., Yaghootkar, H., Schurmann, C., Kettunen, J., Christiansen, M.W., Fairfax, B.P., Schramm, K., Powell, J.E., *et al.* (2013). Systematic identification of trans eQTLs as putative drivers of known disease associations. *Nat Genet* **45**, 1238–1243.

Whitehouse, A.J.O., Bishop, D.V.M., Ang, Q.W., Pennell, C.E., & Fisher, S.E. (2011) CNTNAP2 variants affect early language development in the general population. *Genes Brain Behav* **10**, 451–456.

Wigg, K.G., Couto, J.M., Feng, Y., Anderson, B., Cate-Carter, T.D., Macciardi, F., Tannock, R., Lovett, M.W., Humphries, T.W., & Barr, C.L. (2004) Support for EKN1 as the susceptibility locus for dyslexia on 15q21. *Mol Psychiatry* **9**, 1111–1121.

Wilcke, A., Weissfuss, J., Kirsten, H., Wolfram, G., Boltze, J., & Ahnert, P. (2009) The role of gene DCDC2 in German dyslexics. *Ann Dyslexia* **59**, 1–11.

Wilcke, A., Ligges, C., Burkhardt, J., Alexander, M., Wolf, C., Quente, E., Ahnert, P., Hoffmann, P., Becker, A., Müller-Myhsok, B., *et al.* (2012) Imaging genetics of FOXP2 in dyslexia. *Eur J Hum Genet* **20**, 224–229.

Zeller, T., Wild, P., Szymczak, S., Rotival, M., Schillert, A., Castagne, R., Maouche, S., Germain, M., Lackner, K., Rossmann, H., et al. (2010). Genetics and beyond--the transcriptome of human monocytes and disease susceptibility. *PLoS One* **5**, e10693.
